# Supplementary material for: BRAFV600E-mutated serrated colorectal neoplasia drives transcriptional activation of cholesterol metabolism
Source: Commun Biol. 2023 Sep 21;6:962. doi: 10.1038/s42003-023-05331-x (PMC10514332; doi:10.1038/s42003-023-05331-x)
Supplement: Supplementary file 2 — Supplementary Information [file 42003_2023_5331_MOESM2_ESM.pdf]

BRAF^{V600E}-mutated serrated colorectal neoplasia drives transcriptional activation of cholesterol metabolism.

Paulina Rzasa[1†], Sarah Whelan[1†], Pooyeh Farahmand[1†], Hong Cai[1], Inna Guterman[1], Raquel Palacios-Gallego[1], Shanthi S Undru[1], Lauren Sandford[1ξ], Caleb Green[1], Catherine Andreadi[1], Maria Mintseva[1,2], Emma Parrott[1], Hong Jin[1], Fiona Hey[1], Susan Giblett[1], Nicolas B Sylvius[3], Natalie S Allcock[4], Anna Straatman-Iwanowska[4], Roberto Feuda[5], Cristina Tufarelli[1], Karen Brown[1], Catrin Pritchard[1] and Alessandro Rufini[1,6].

[1]Leicester Cancer Research Centre, University of Leicester, Leicester, UK.

[2]Area of Neuroscience, International School for Advanced Studies (SISSA), Trieste, Italy.

[3]NUCLEUS Genomics, Core Biotechnology Services, University of Leicester, Leicester, UK.

[4]University of Leicester Core Biotechnology Services Electron Microscopy Facility, Leicester, UK.

[5]Department of Genetics and Genome Biology, University of Leicester, Leicester, UK.

[6]Dipartimento di Bioscienze, University of Milan, Milan, Italy.

[ξ] Current address: Institute of Cancer and Genomic Sciences, University of Birmingham, Birmingham, UK.

[†]These authors equally contributed to this work.

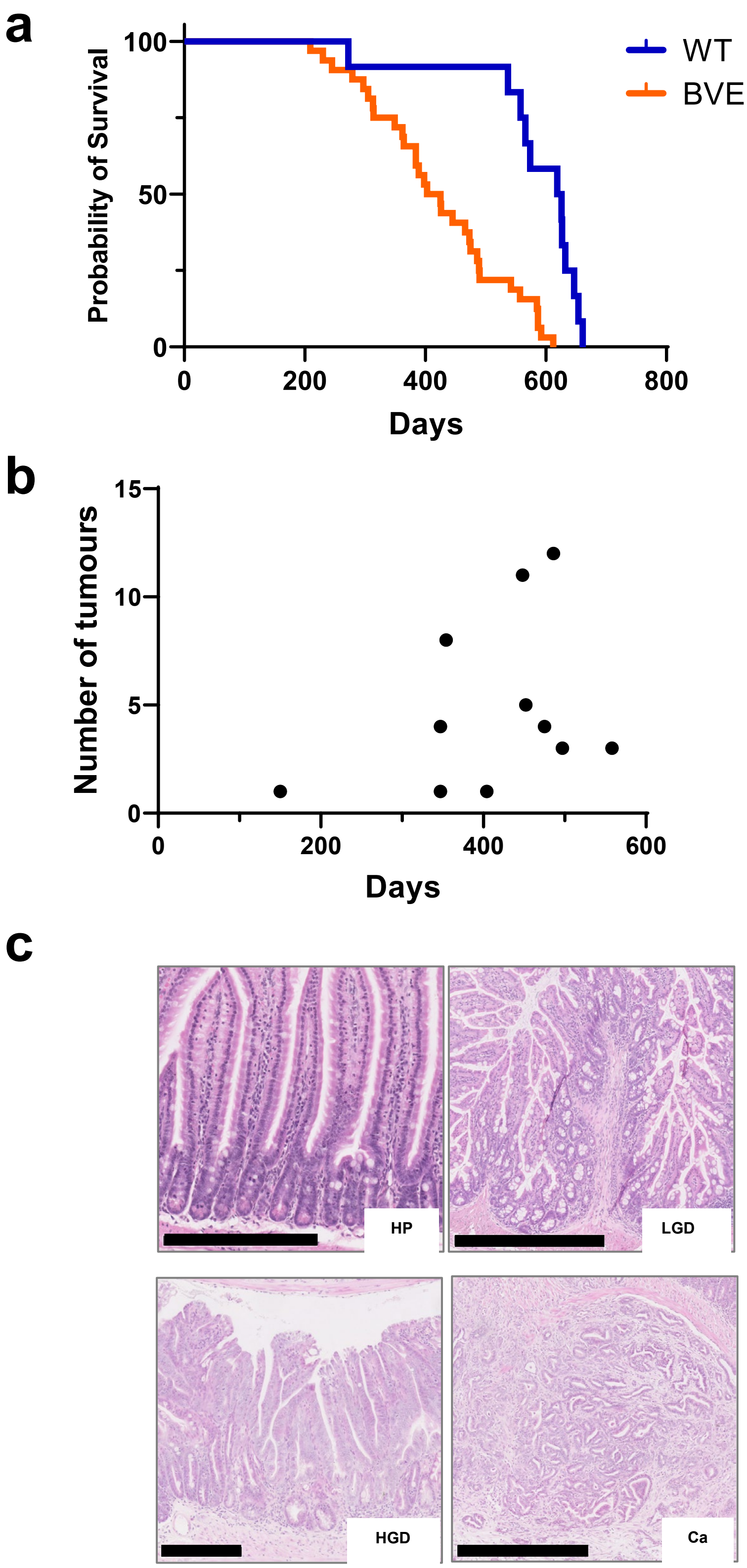

Supplementary Figure 1. Expression of *Braf*^{V600E} in the intestinal epithelium reduces animal lifespan and generates limited tumorigenesis. a) Kaplan-Meyer survival analysis showing reduced survival of *Braf*^{V600E} mice (median survival 622 days and 414 days, $p < 0.0001$ Mantel-Cox Log-rank test). **b)** Graph showing the number of tumors in mice culled at the indicated time post-tamoxifen injection. **c)** H&E representative images of intestinal tissue hyperplasia (HP) and intestinal lesions. LGD=low grade dysplasia, HGD=high grade dysplasia, Ca=cancer. Scale bar= 250 μm for HP and HGD and 500 μm for LGD and Ca.

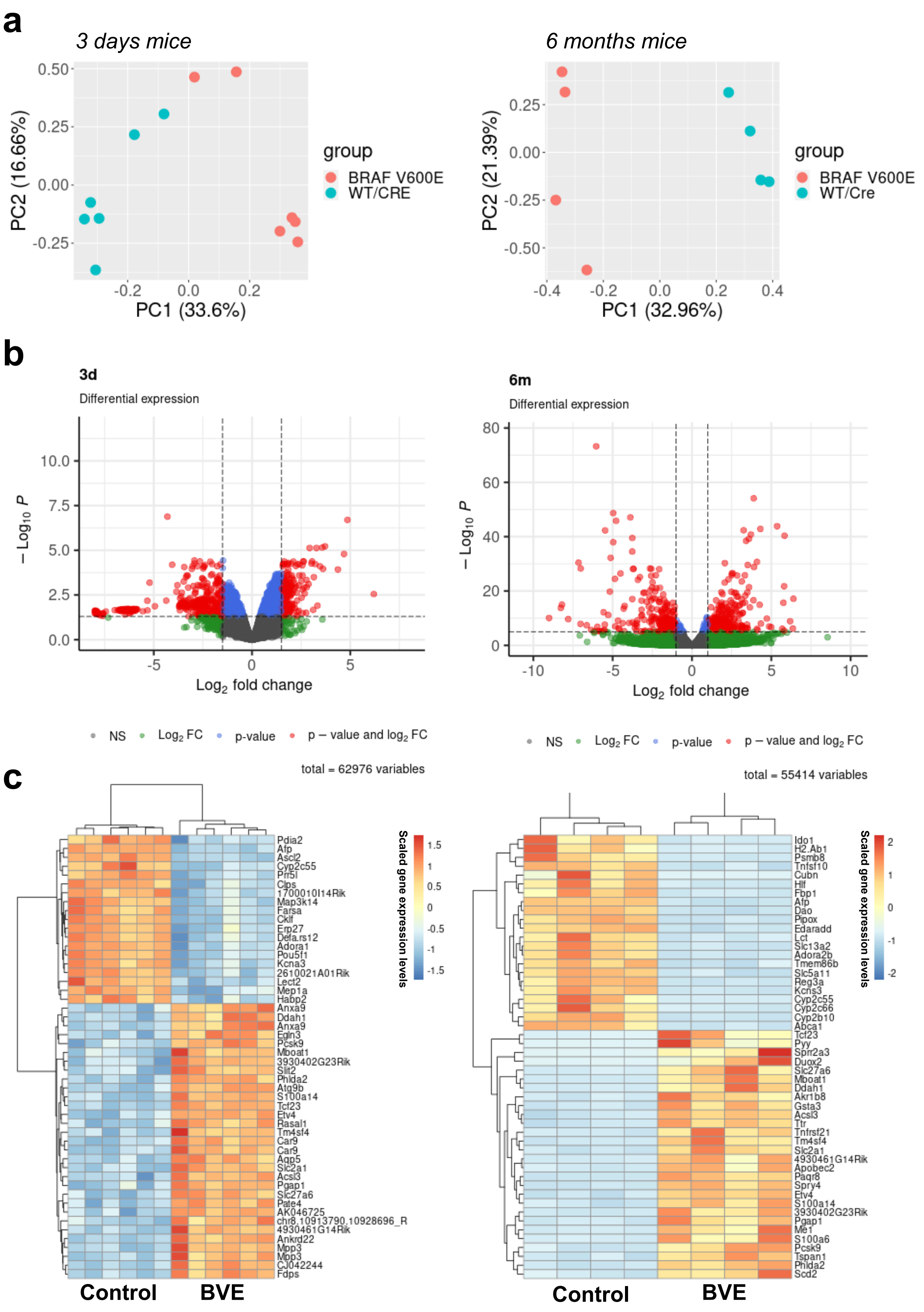
a
3 days mice
PC2 (16.66%)
PC1 (33.6%)
0.50
0.25
0.00
-0.25
-0.2
0.0
0.2
group
BRAF V600E
WT/CRE
6 months mice
PC2 (21.39%)
PC1 (32.96%)
0.25
0.00
-0.25
-0.50
-0.4
-0.2
0.0
0.2
0.4
group
BRAF V600E
WT/Cre
b
3d
Differential expression
$-\text{Log}_{10}\ P$
10.0
7.5
5.0
2.5
0.0
-5
0
5
Log_2 fold change
NS
Log_2 FC
p-value
p – value and log_2 FC
total = 62976 variables
6m
Differential expression
$-\text{Log}_{10}\ P$
80
60
40
20
0
-10
-5
0
5
10
Log_2 fold change
NS
Log_2 FC
p-value
p – value and log_2 FC
total = 55414 variables
c
Pdia2
Afp
Ascl2
Cyp2c55
Prr5l
Clps
1700010I14Rik
Map3k14
Farsa
Cklf
Erp27
Defa.rs12
Adora1
Pou5f1
Kcna3
2610021A01Rik
Lect2
Mep1a
Habp2
Anxa9
Ddah1
Anxa9
Egln3
Pcsk9
Mboat1
3930402G23Rik
Slit2
Phlda2
Atg9b
S100a14
Tcf23
Etv4
Rasal1
Tm4sf4
Car9
Car9
Aqp5
Slc2a1
Acsl3
Pgap1
Slc27a6
Pate4
AK046725
chr8.10913790.10928696_R
4930461G14Rik
Ankrd22
Mpp3
Mpp3
CJ042244
Fdps
Scaled gene expression levels
1.5
1
0.5
0
-0.5
-1
-1.5
Control
BVE
Ido1
H2.Ab1
Psmb8
Tnfsf10
Cubn
Hlf
Fbp1
Afp
Dao
Pipox
Edaradd
Lct
Slc13a2
Adora2b
Tmem86b
Slc5a11
Reg3a
Kcns3
Cyp2c55
Cyp2c66
Cyp2b10
Abca1
Tcf23
Pyy
Sprr2a3
Duox2
Slc27a6
Mboat1
Ddah1
Akr1b8
Gsta3
Acsl3
Ttr
Tnfrsf21
Tm4sf4
Slc2a1
4930461G14Rik
Apobec2
Paqr8
Spry4
Etv4
S100a14
3930402G23Rik
Pgap1
Me1
S100a6
Pcsk9
Tspan1
Phlda2
Scd2
Scaled gene expression levels
2
1
0
-1
-2
Control
BVE

Supplementary Figure 2. Transcriptomic analysis of mice after short term (3 days) and long-term (6 months) induction of the *Braf^V600E^* oncogene. a) Principal component analysis (PCA) of *Braf*^{V600E} mice and WT controls showing clustering by genotype. n=6 animals per genotype. **b)** Volcano plot of differentially expressed genes in *Braf*^{V600E} versus WT control mice. Log2 for fold change in expression on the X-axis and Log10 for adjust p value on Y-axis. Results are color coded: adjusted p value less than 0.05 (grey), log2 fold change greater than 0.5 (green), adjusted p value less than 0.05 (blue), and both adjusted p value less than 0.05 and log2 fold change greater than 0.5 (red). **c)** Heat map with the most top significantly up and down regulated genes in *Braf*^{V600E} intestine.

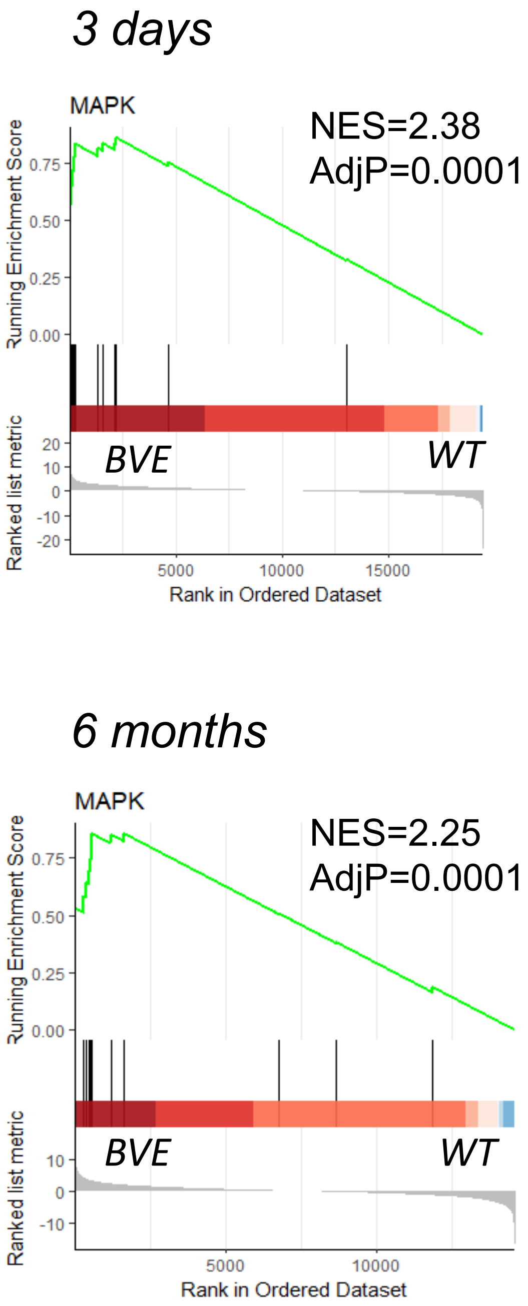

Supplementary Figure 3. GSEA showing persistent enrichment of a MAPK signature in the intestinal tissue of *Braf*^{V600E} mice. NES = normalized enrichment score.

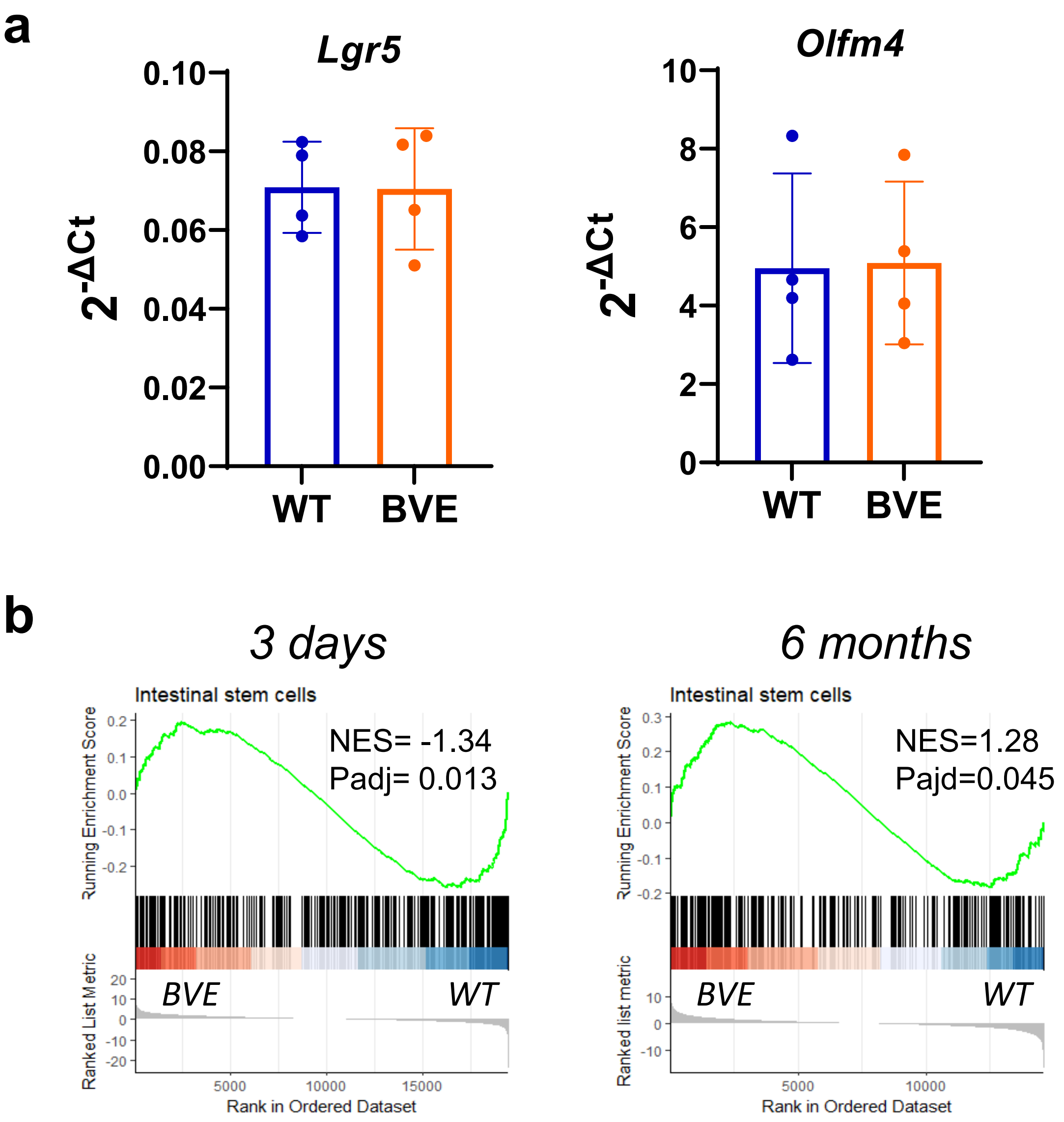

Supplementary Figure 4. Persistence of ISCs following expression of *Braf*^{V600E} in intestinal tissue. a) rt-qPCR analysis of intestinal stem cells biomarkers *Lgr5* and *Olfm4* 6 months after tamoxifen injection. Bar graphs represent mean ± SD. Each dot represents a single mouse. Data were analyzed by unpaired two-tailed t-test (n=4 per group). ns = not significant. **b)** GSEA 3 days and 6 months after induction of mutant *Braf* showing changes in the expression of an intestinal stem cells signature. NES = normalized enrichment score.

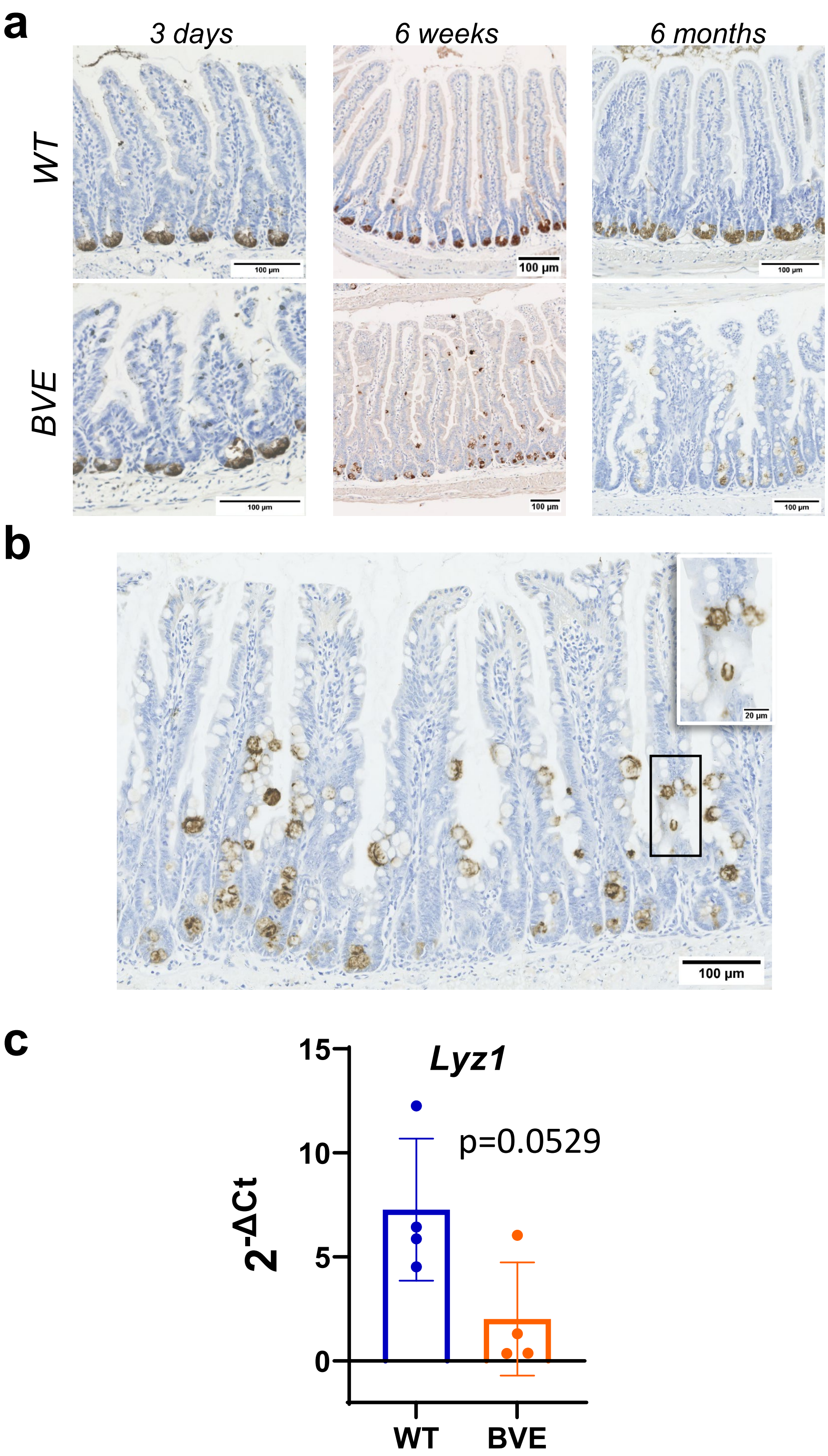

Supplementary Figure 5. Expression of *Braf*^{V600E} in the intestinal epithelium alters homeostasis of Paneth cells. a) Representative histological images of small intestinal tissue stained with the lysozyme marker from WT mice and *Braf*^{V600E} mice (BVE) at 3 days, 6 weeks and 6 months following tamoxifen induction. Bar size = 100 µm. **b)** Details of delocalized Paneth cells in 6-month induced mutant mice. Bar size = 100 µm (20 µm inset). **c)** rt-qPCR analysis of the expression of the Paneth cell marker Lysozyme 6 months after induction of *Braf*^{V600E}. Data are plotted as mean ± SD. Each dot represents a single mouse. Data were analyzed by unpaired two-tailed t-test (n=4 per group).

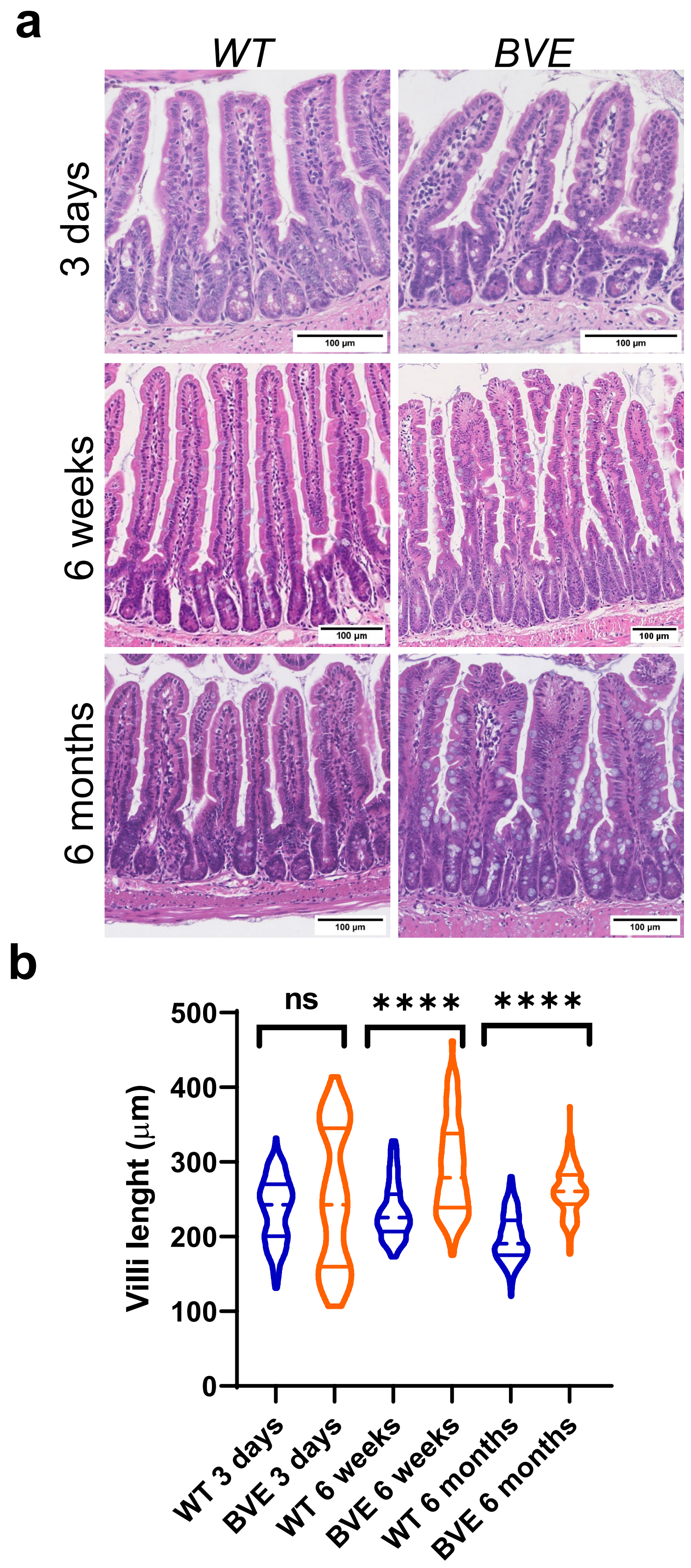

Supplementary Figure 6. Expression of *Braf*^{V600E} in the intestinal epithelium alters intestinal tissue homeostasis. a) Representative histological images of small intestinal tissue from WT and *Braf*^{V600E} mice (BVE) at 3 days, 6 weeks and 6 months following tamoxifen induction. Bar size = 100 µm. **b)** Violin plot showing quantification of villi length 3 days, 6 weeks and 6 months following tamoxifen induction. Dotted and solid lines indicate median and quartiles, respectively. Data were analyzed by unpaired, two-tailed t-test (n=3 per group). ns = no significant, ***P ≤ 0.001, ****P ≤ 0.0001.

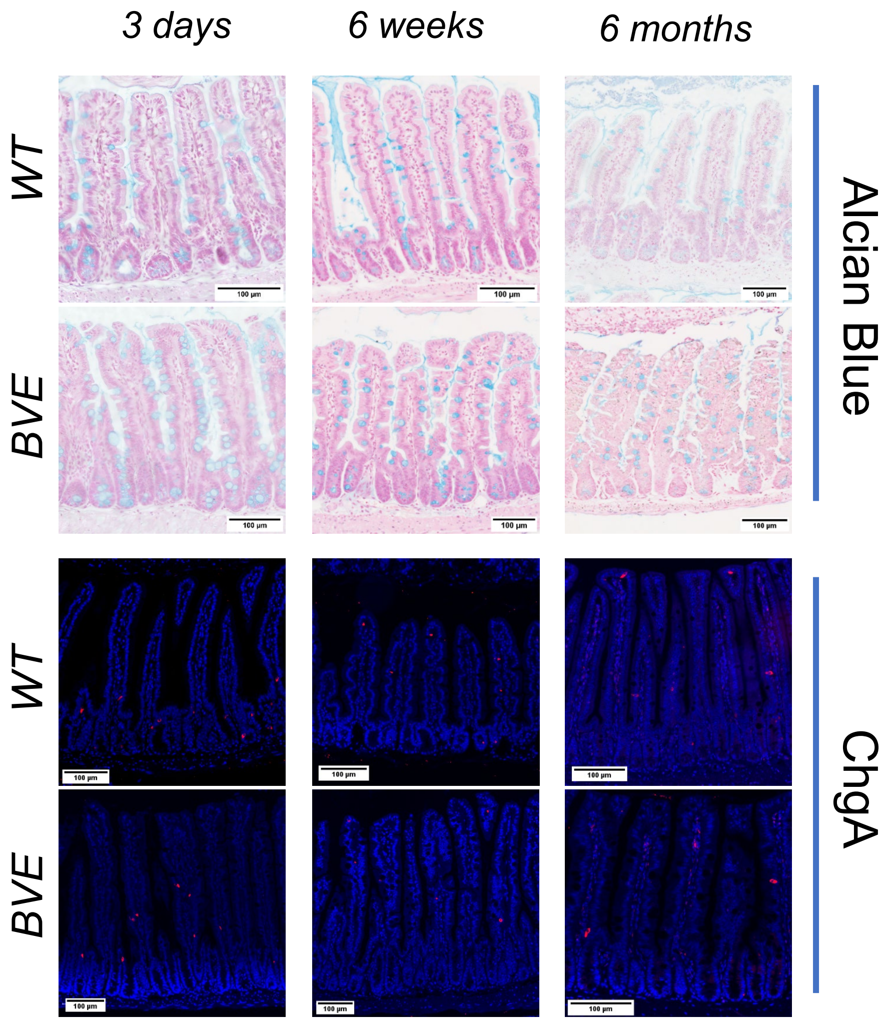

Supplementary Figure 7. Expression of *Braf*^{V600E} in the intestinal epithelium alters homeostasis of EE cells. Representative histological images of small intestinal tissue stained with Alcian Blue (Goblet cells) and the EEC marker ChgA in the intestinal tissue of mice with the indicated genotypes. Independent WT mice were used for each different time point, but, for ease of reference, representative examples from 6 months mice are shown. Bar size = 100 μm.

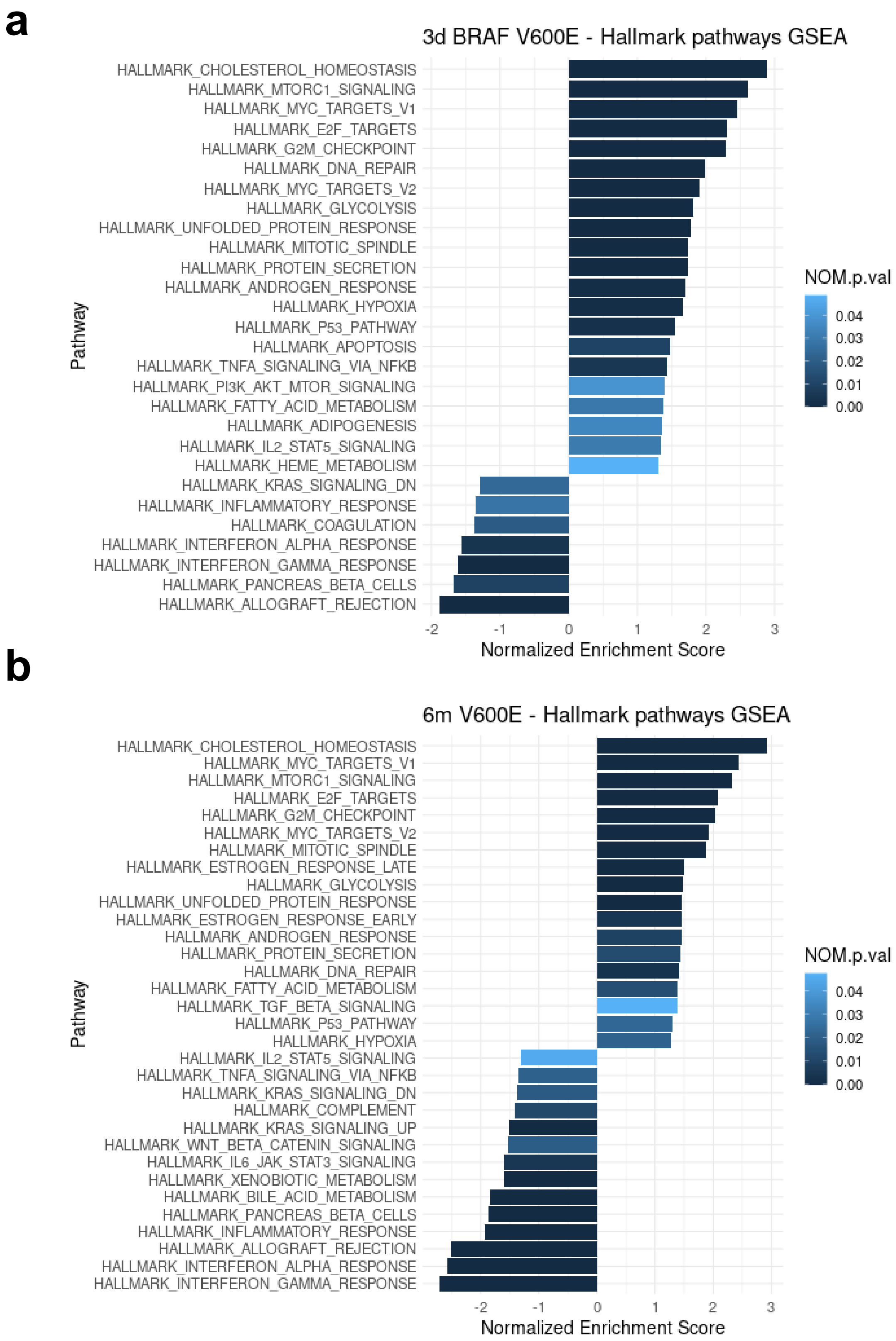

Supplementary Figure 8. GSEA of mouse transcriptomic data. a) and **b)** most significant up and down regulated signatures from the MSigDB in *Braf*^{V600E} mice at 3 days (a) and 6 months (b) following induction of mutant *Braf*.

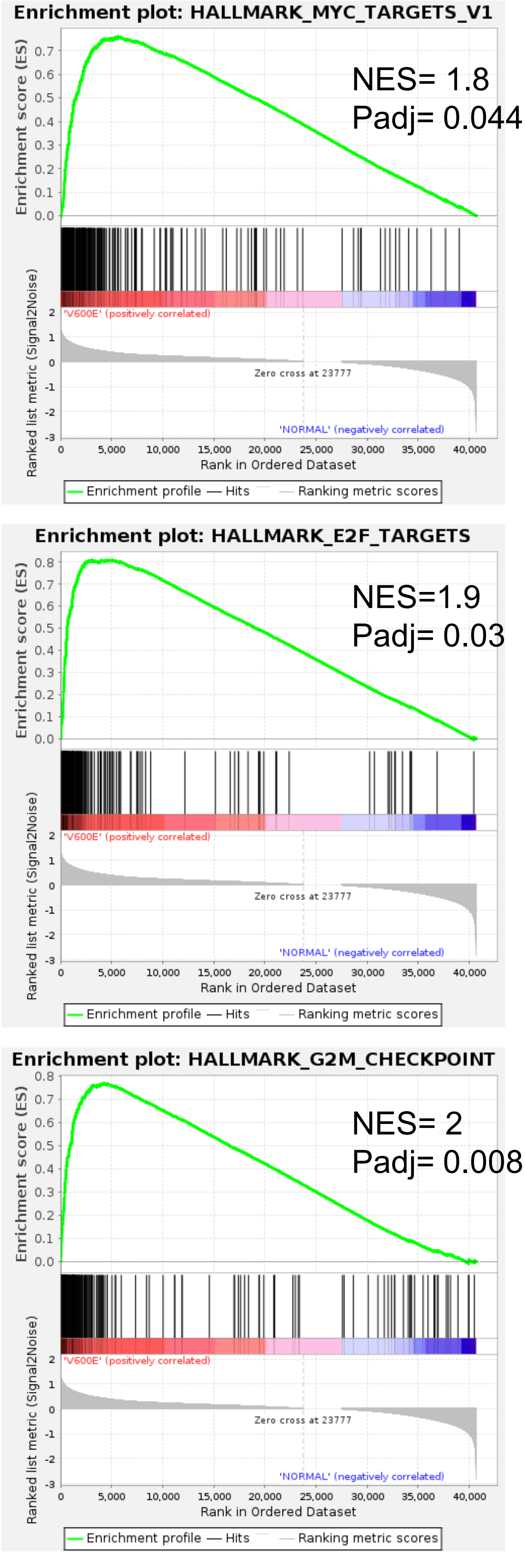

Supplementary Figure 9. Analysis of the TCGA transcriptomic dataset. GSEA analysis shows significant enrichment of the Myc targets, E2F targets and G2M checkpoint signatures. NES = normalized enrichment score.

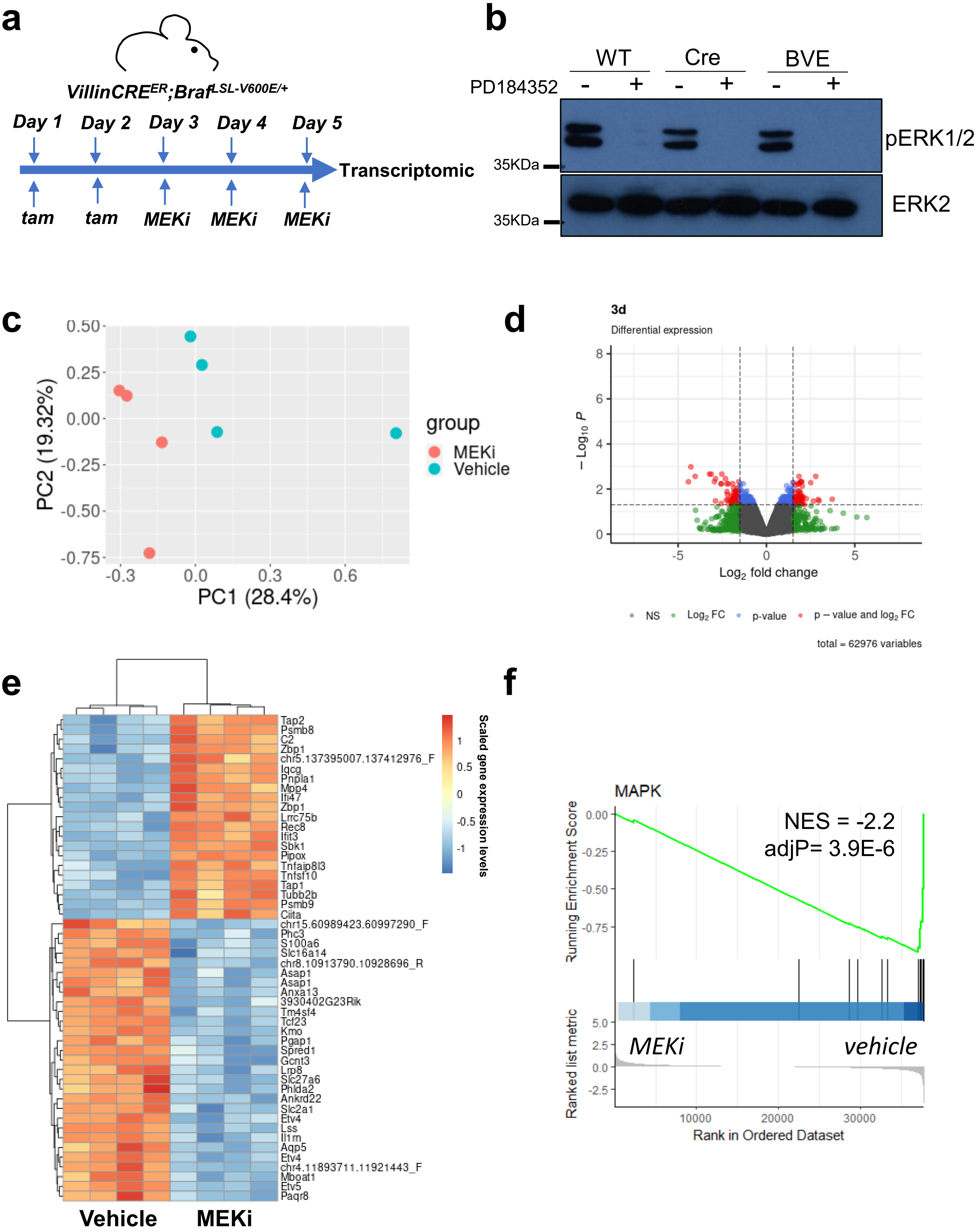
a
VillinCRE^ER;Braf^LSL-V600E/+
Day 1
Day 2
Day 3
Day 4
Day 5
Transcriptomic
tam
tam
MEKi
MEKi
MEKi
b
WT
Cre
BVE
PD184352
- + - + - +
pERK1/2
ERK2
35KDa
35KDa
c
PC2 (19.32%)
PC1 (28.4%)
0.50
0.25
0.00
-0.25
-0.50
-0.75
-0.3
0.0
0.3
0.6
group
MEKi
Vehicle
d
3d
Differential expression
– Log10 P
Log2 fold change
8
6
4
2
0
-5
0
5
NS
Log2 FC
p-value
p – value and log2 FC
total = 62976 variables
e
Tap2
Psmb8
C2
Zbp1
chr5.137395007.137412976_F
Iqcg
Pnpla1
Mpp4
Ifi47
Zbp1
Lrrc75b
Rec8
Ifit3
Sbk1
Pipox
Tnfaip8l3
Tnfsf10
Tap1
Tubb2b
Psmb9
Ciita
chr15.60989423.60997290_F
Phc3
S100a6
Slc16a14
chr8.10913790.10928696_R
Asap1
Asap1
Anxa13
3930402G23Rik
Tm4sf4
Tcf23
Kmo
Pgap1
Spred1
Gcnt3
Lrp8
Slc27a6
Phlda2
Ankrd22
Slc2a1
Etv4
Lss
Il1rn
Aqp5
Etv4
chr4.11893711.11921443_F
Mboat1
Etv5
Paqr8
Scaled gene expression levels
1
0.5
0
-0.5
-1
Vehicle
MEKi
f
MAPK
NES = -2.2
adjP= 3.9E-6
Running Enrichment Score
0.00
-0.25
-0.50
-0.75
Ranked list metric
5.0
2.5
0.0
-2.5
MEKi
vehicle
10000
20000
30000
Rank in Ordered Dataset

Supplementary Figure 10. Inhibition of MAPK pathway with the MEK inhibitor PD1843352 (MEKi). a) Schematic of the experimental design for *in vivo* inhibition of the MAPK pathway using MEKi. **b)** Western blot analysis of phosphorylated Erk protein in intestinal tissue showing efficient inhibition of the MAPK pathway in mice treated with MEKi. **c)** Principal component analysis (PCA) of *Braf*^{V600E} mice treated with MEKi or vehicle. n=6 animals per genotype. **d)** Volcano plot of differentially expressed genes in *Braf*^{V600E} treated with MEKi versus vehicle control mice. Log2 for fold change in expression on the X-axis and Log10 for adjust p value on Y-axis. Results are color coded: adjusted p value less than 0.05 (grey), log2 fold change greater than 0.5 (green), adjusted p value less than 0.05 (blue), and both adjusted p value less than 0.05 and log2 fold change greater than 0.5 (red). **e)** Heat map with the most top significantly up and down regulated genes in *Braf*^{V600E} mice treated with MEKi. **f)** GSEA showing downregulation of MAPK pathway signature in mice expressing *Braf*^{V600E} for 3 days and treated with the MEK MEKi. NES = normalized enrichment score.

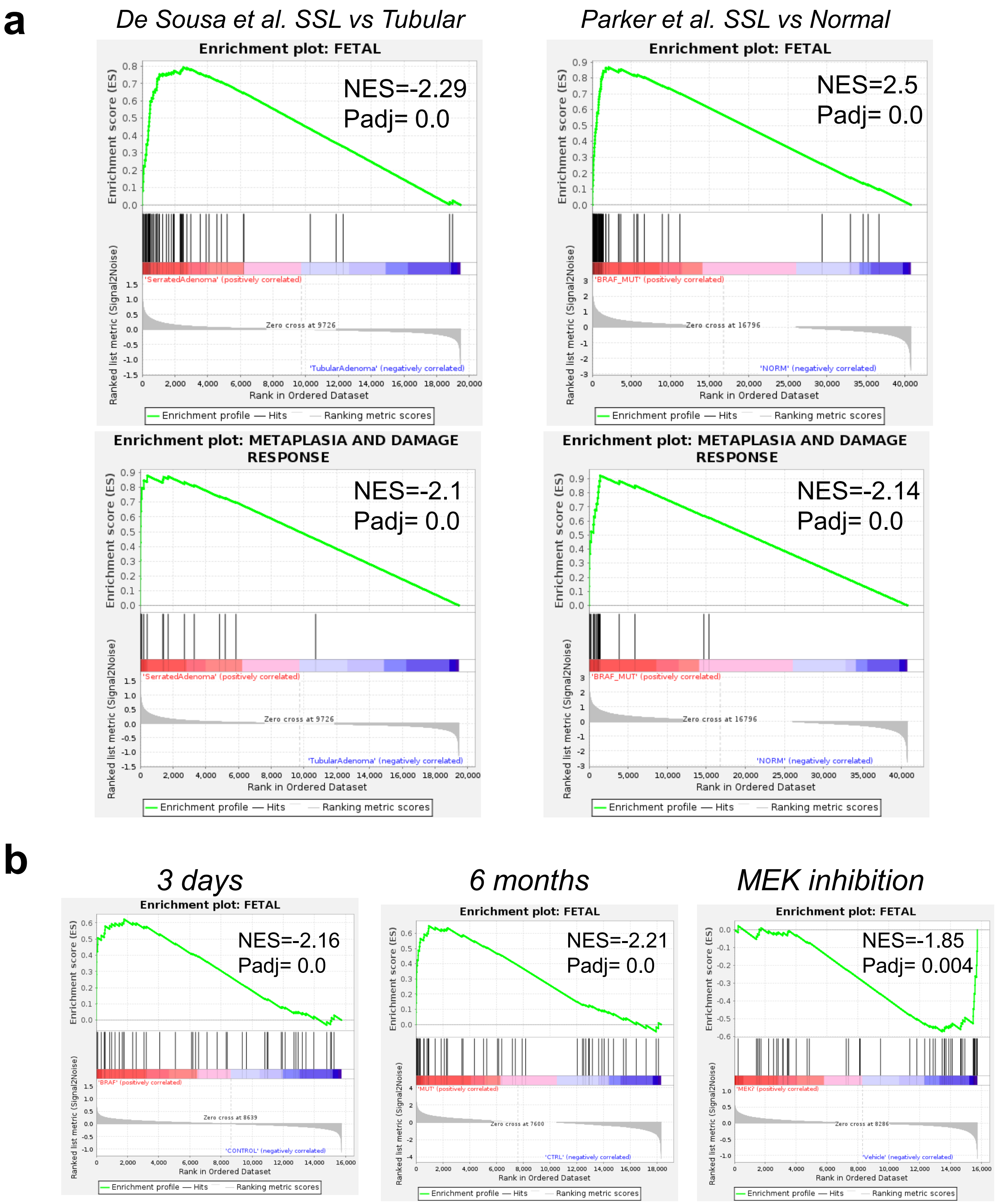

Supplementary Figure 11. Expression of *Braf*^{V600E} in the mouse intestinal epithelium leads to enrichment of a fetal-like gene signature. A) GSEA of the fetal and metaplasia gene signatures in two independent human dataset comparing SSLs to tubular adenomas or matched normal tissue. **B)** GSEA of the fetal signature the intestinal tissue of *Braf*^{V600E} mice 3 days and 6 months following induction of mutant *Braf*. Dependence on MAPK is also shown. NES = normalized enrichment score.

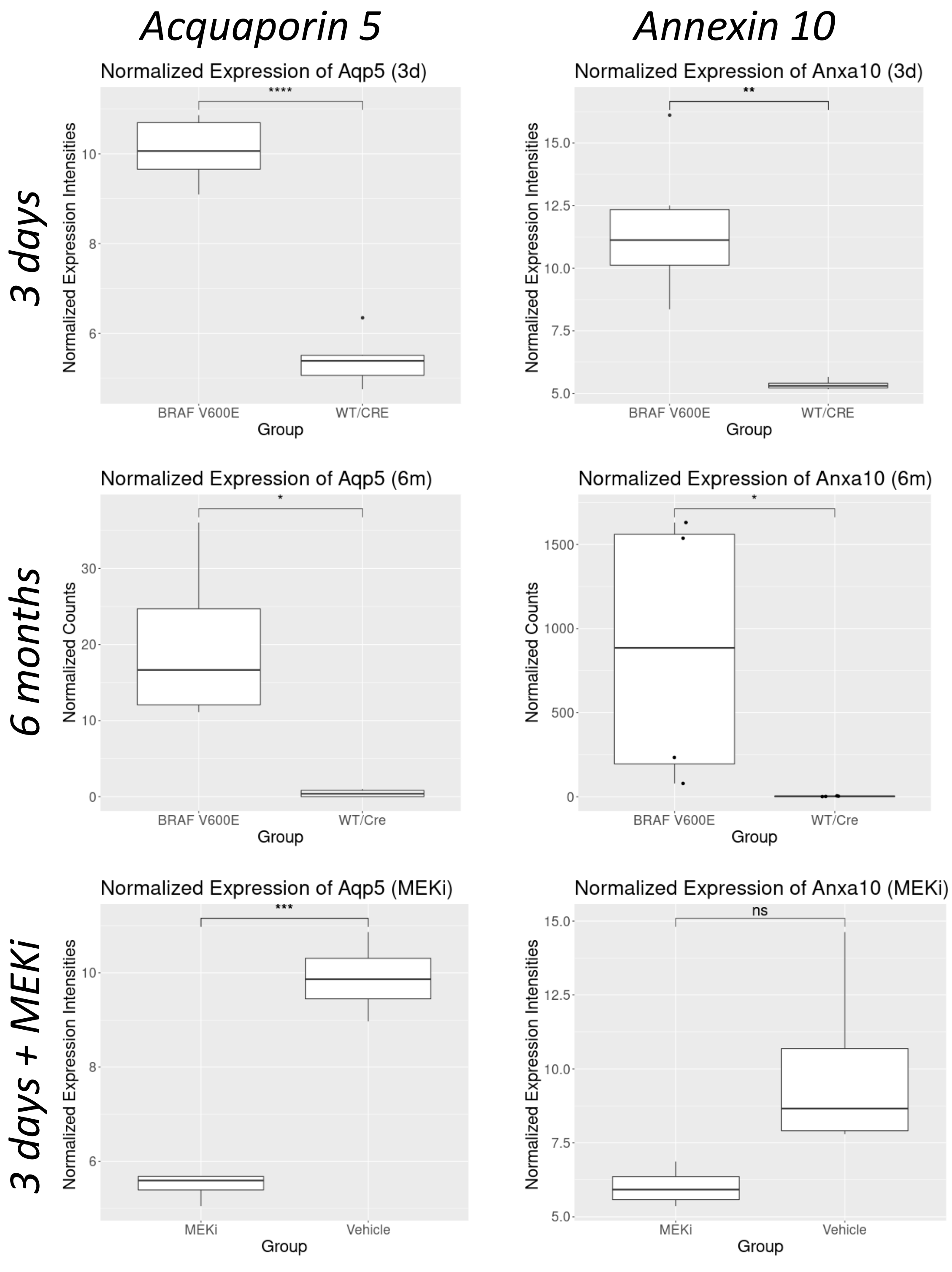

Supplementary Figure 12. Expression of metaplastic genes *Aqp5* and *Anxa10* in intestinal tissue of mice with the *Braf*^{V600E} mutation. Normalized expression levels of the *Aqp5* and *Anxa10* genes in the intestinal tissue at the indicated time points. Dependence on MEKi is also shown. Data are plotted as box and whiskers plots. The bars within the box represent the median value, whiskers represent min and max value. The p value was determined using Wald test. ns = not significant, *p ≤ 0.05, **p ≤ 0.01, ***p ≤ 0.001, ****p ≤ 0.0001.

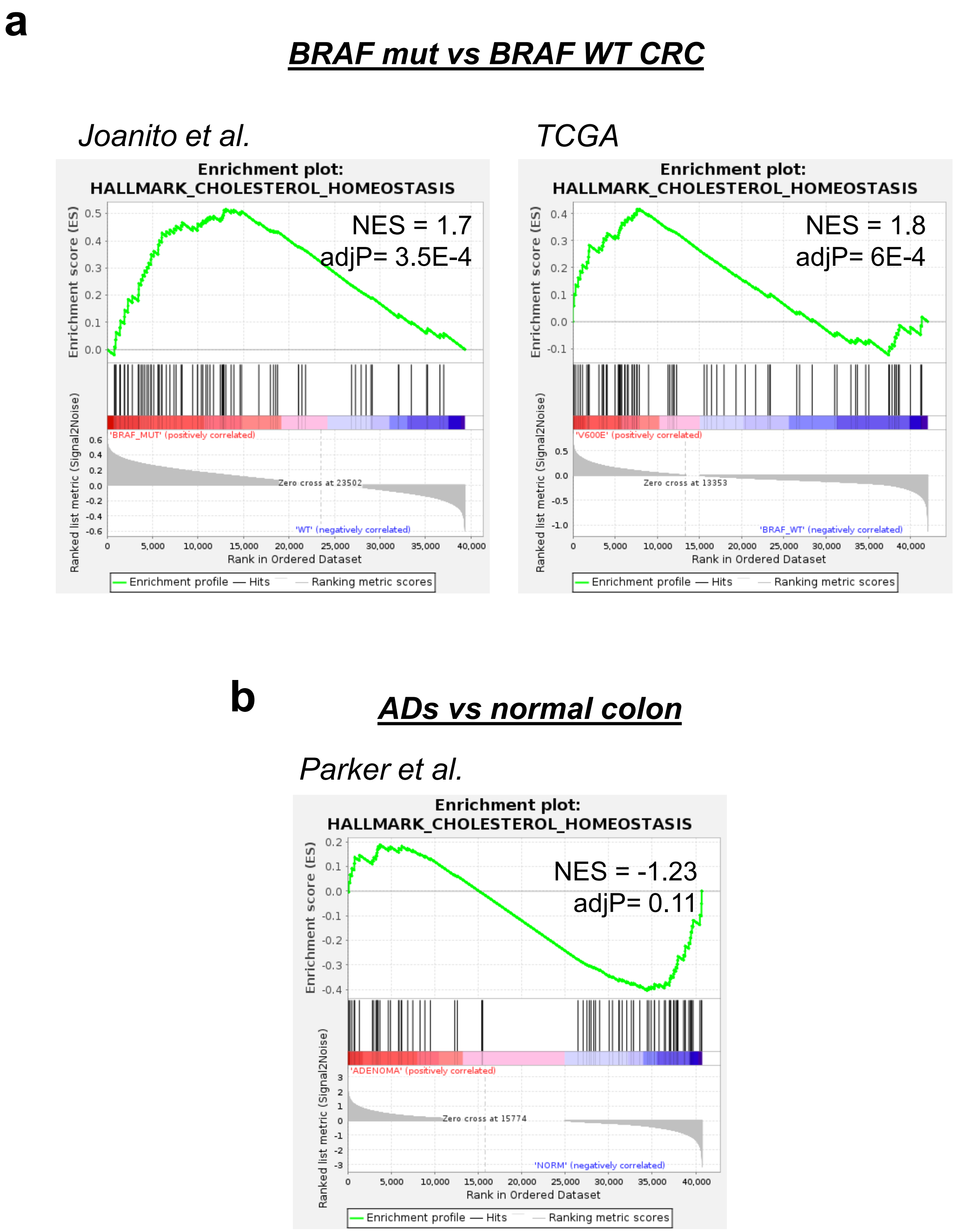

Supplementary Figure 13. Analysis of human transcriptomic datasets. A) GSEA shows enrichment of the cholesterol gene signature in CRC harboring mutant *BRAF* in two independent datasets from Joanito et al. (n=15 mutant and 147 WT cases) and from the TCGA dataset (n=35 mutant and 236 WT cases). **B)** GSEA shows lack of transcriptional enrichment of the cholesterol gene signature in adenomas lesions compared to match normal tissue.

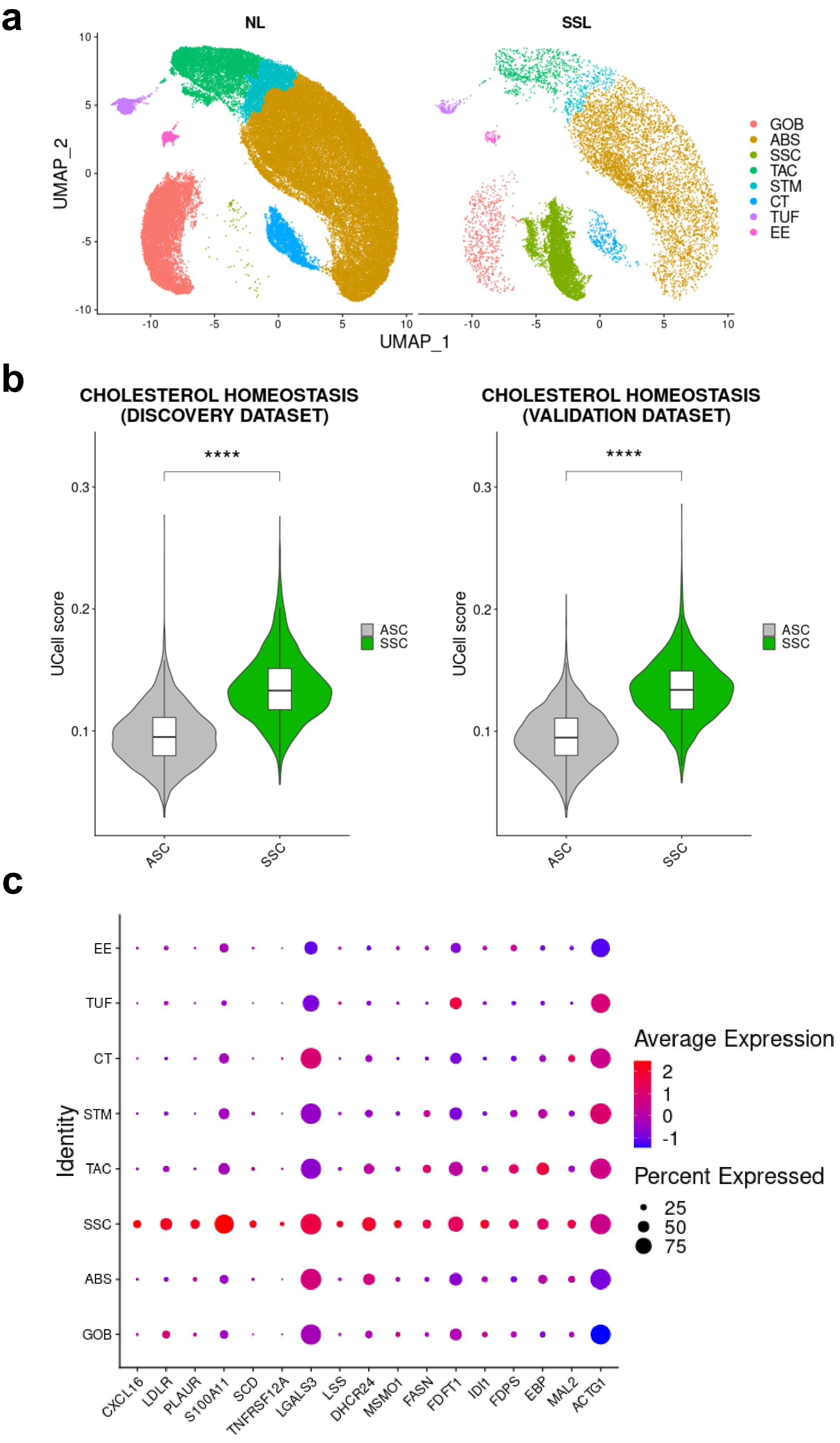
a
NL
SSL
UMAP_2
UMAP_1
10
5
0
-5
-10
GOB
ABS
SSC
TAC
STM
CT
TUF
EE
b
CHOLESTEROL HOMEOSTASIS (DISCOVERY DATASET)
CHOLESTEROL HOMEOSTASIS (VALIDATION DATASET)
****
UCell score
0.3
0.2
0.1
ASC
SSC
c
Identity
EE
TUF
CT
STM
TAC
SSC
ABS
GOB
CXCL16
LDLR
PLAUR
S100A11
SCD
TNFRSF12A
LGALS3
LSS
DHCR24
MSMO1
FASN
FDFT1
IDI1
FDPS
EBP
MAL2
ACTG1
Average Expression
2
1
0
-1
Percent Expressed
25
50
75

Supplementary Figure 14. Single cells analysis of cholesterol biosynthesis in SSLs. a) UMAP representation of epithelial scRNA-seq data, color-coded by cell type and comparing cell population in, normal tissue (NL), SSL, sessile serrated lesions. ABS, absorptive cells; CT, crypt top colonocytes; EE, enteroendocrine cells; GOB, goblet cells; SSC, serrated specific cells; STM, stem cells; TAC, transient amplifying cells; TUF, tuft cells. **b)** Violin plot comparing UCell score distribution of a cholesterol gene signature between SSC and ASC cell populations in DIS (left) and VAL (right) datasets. The boxes represent median and interquartile ranges. The whiskers show 95% confidence interval. Distribution of UCell score was compared using Wilcoxon test. ****p ≤ 0.0001. SSC, serrated specific cells; ASC, adenoma specific cells. **c)** Dot plot of genes within the cholesterol signature showing significant upregulation in gene expression between SSC and non-tumor cells. Dot color indicates average gene expression levels, dot size indicates the percentage of cells within a population that express the indicated gene.

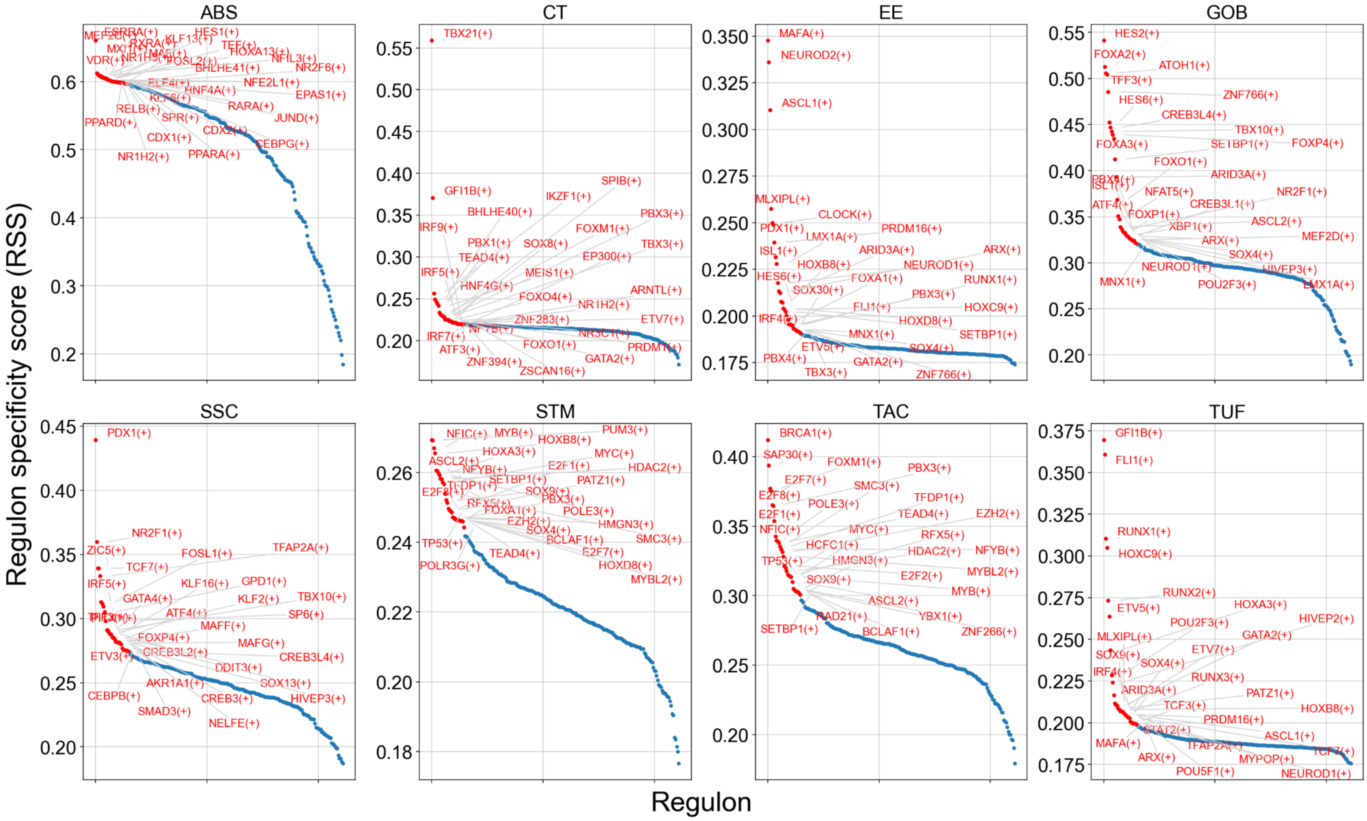

Supplementary Figure 15. Analysis of regulons in SSLs. Plot of the top 30 specific regulons (highlighted in red) in the indicated cell lines from analysis of single cell data of SSL and NL dataset. The regulon specificity score (RSS) is shown on y axis. ABS, absorptive cells; CT, crypt top colonocytes; EE, enteroendocrine cells; GOB, goblet cells; SSC, serrated specific cells; STM, stem cells; TAC, transient amplifying cells; TUF, tuft cells.

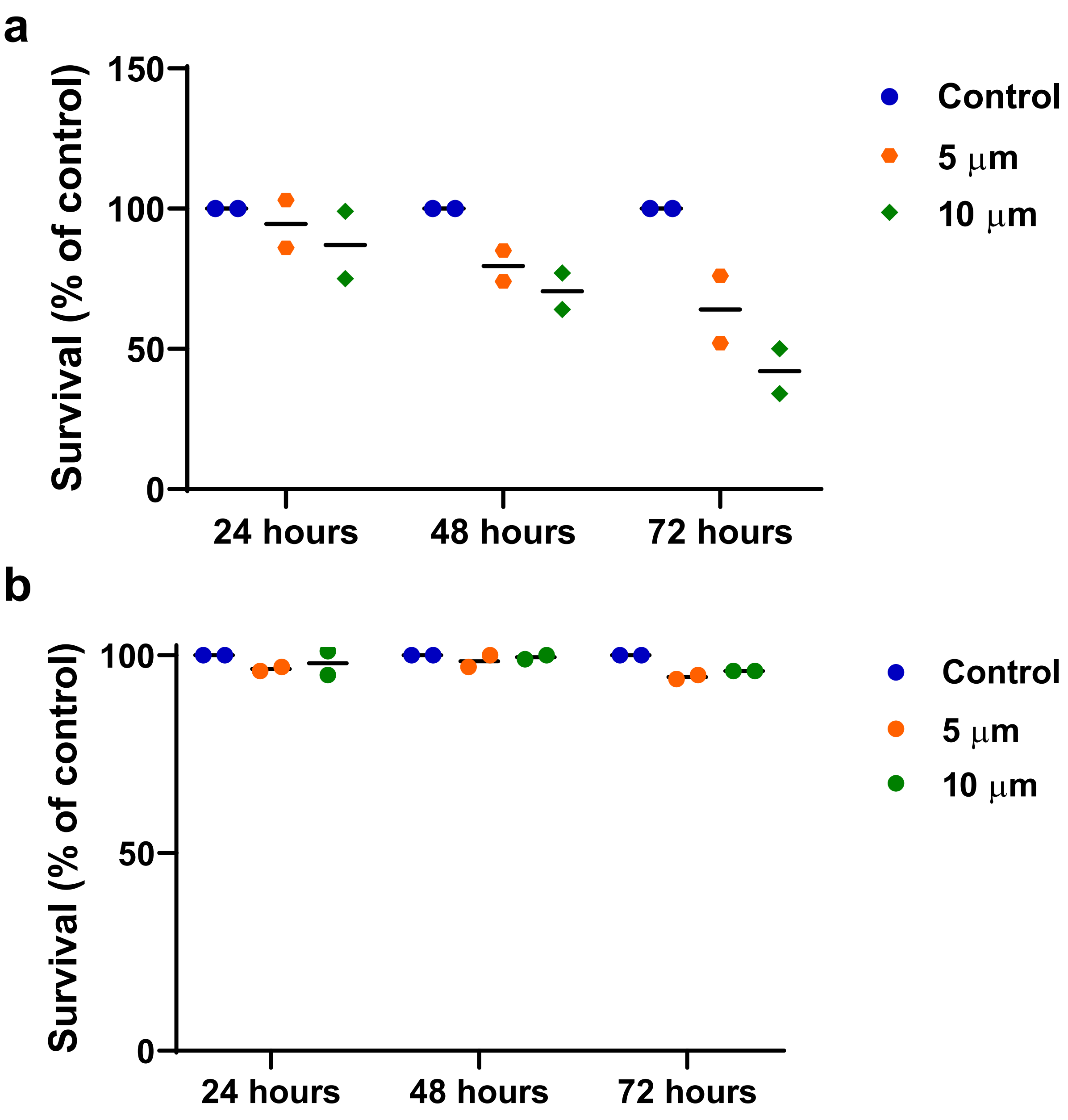

Supplementary Figure 16. The effects of cholesterol biosynthesis inhibition on cell viability in *BRAF*^{V600E} CRC cells. Dot plot showing the effect of different concentrations (0, 5 and 10 μM) of atorvastatin and periods of treatment (24, 48 and 72 hours) on RKO (a) and HT-29 (b) cell lines. Values were normalized against the control mean by dividing the measured optical density by the mean control optical density and multiplying by 100 to give cell viability as a percentage of the control. Results were obtained from two independent experiments. Lines show average value of duplicates..

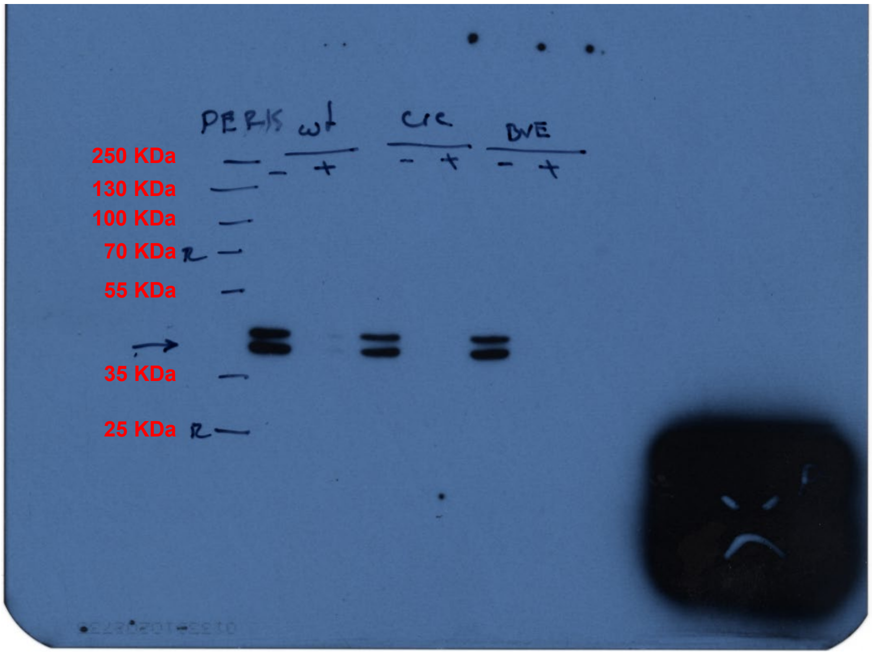

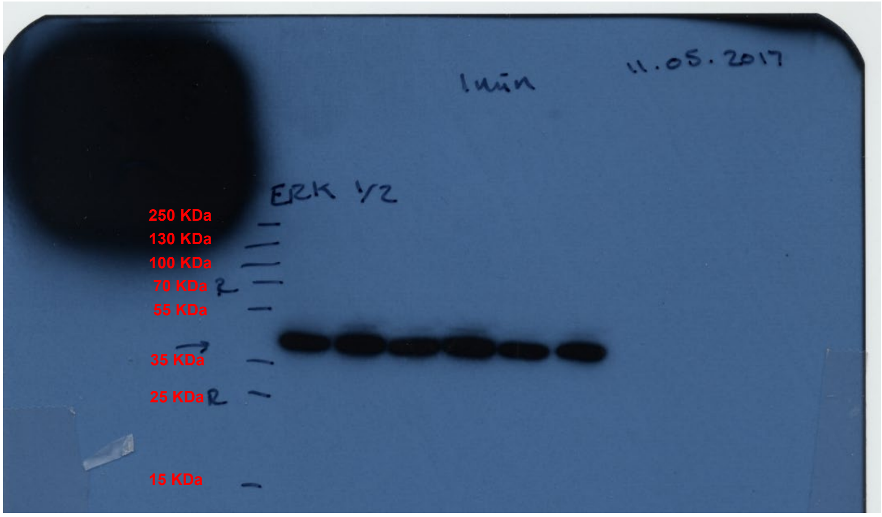

Supplementary Figure 17. Original western blots. Original uncropped western blot images related to Supplementary Figure 10B. The molecular weight sizes are predicted, as we could no capture the pre-stained ladder on the film.

Target	Primers	Sequence
**BRAFLSL-V600E/WT**	OCP125 (FWD)	5’-GCC-CAG-GCT-CTT-TAT-GAG-AA-3’
	OCP137 (REV-HET)	5’-GCT-TGG-CTG-GAC-GTA-AAC-TC-3’
	OCP 143 (REV-WT)	5’-AGT-CAA-TCA-TCC-ACA-GAG-ACC-T-3’
**Villin-CreER^{T}**	OCP 361 (FWD)	5’-GCC-TGG-TCT-GGA-CAC-ATG-CC-3’
	OCP362 (REV)	5’-GTG-TCA-GCA-TCC-AAC-AAG-GC-3’

Supplementary Table 1. PCR primers used for genotyping.

Target	Supplier	Catalogue	Species	Dilution	Antigen retrieval
**Lysozyme**	DAKO	A0099	Rabbit	1:10,000	Proteinase K
**Olfm4**	Cell Signalling	39141	Rabbit	1:300	Sodium citrate
**Chromogranin A**	Immunostar	20085		1:1000	Sodium Citrate
**BrdU**	Cell Signalling	5292	Mouse	1:200	Sodium Citrate
**Cleaved-PARP**	Cell Signalling	94885	Rabbit (mono)	1:100	Sodium Citrate

Supplementary Table 2. List of primary antibodies used for IHC and IF.

Name	Fluorophore	Amplicon size (bp)	Assay ID
Lgr5	FAM	64	Mm00438890_m1
Olfm4	FAM	130	Mm01320260_m1
Lyz1	FAM	129	Mm00657323_m1
Alpi	FAM	60	Mm01285814_g1
Muc2	FAM	66	Mm01276696_m1
Chga	FAM	59	Mm00514341_m1
Hmgcr	FAM	64	Mm01282499_m1
Mvk	FAM	67	Mm00445773_m1
Mvd	FAM	109	Mm00507014_m1
Tbp	VIC	65	Mm01277042_m1
Actb	VIC	72	Mm04394036_g1
B2m	VIC	77	Mm00437762_m1
Gusb	VIC	71	Mm01197698_m1

Supplementary Table 3. List of TaqMan essay for rt-qPCR.
