# Supplementary material for: BRAFV600E-mutated serrated colorectal neoplasia drives transcriptional activation of cholesterol metabolism
Source: Commun Biol. 2023 Sep 21;6:962. doi: 10.1038/s42003-023-05331-x (PMC10514332; doi:10.1038/s42003-023-05331-x)
Supplement: Supplementary file 3 — Description of Additional Supplementary Files [file 42003_2023_5331_MOESM3_ESM.pdf]

Description of Additional Supplementary Files

File name: Supplementary Data 1

Description: Numerical source data for graphs in Figure 1b-d; Figure 2 e and f; Figure 4c, e and f; Figure 5e

File name: Supplementary Data 2

Description: The numerical source data behind the PARP positive cells in Figure 4g.
