# Supplementary material for: BRAFV600E-mutated serrated colorectal neoplasia drives transcriptional activation of cholesterol metabolism
Source: Commun Biol. 2023 Sep 21;6:962. doi: 10.1038/s42003-023-05331-x (PMC10514332; doi:10.1038/s42003-023-05331-x)
Supplement: Supplementary file 6 — Reporting Summary [file 42003_2023_5331_MOESM6_ESM.pdf]

nature portfolio

Corresponding author(s): Alessandro Rufini

Last updated by author(s): Aug 28, 2023

Reporting Summary

Nature Portfolio wishes to improve the reproducibility of the work that we publish. This form provides structure for consistency and transparency in reporting. For further information on Nature Portfolio policies, see our Editorial Policies and the Editorial Policy Checklist.

Statistics

For all statistical analyses, confirm that the following items are present in the figure legend, table legend, main text, or Methods section.

n/a	Confirmed	
☐	☒	The exact sample size (*n*) for each experimental group/condition, given as a discrete number and unit of measurement
☐	☒	A statement on whether measurements were taken from distinct samples or whether the same sample was measured repeatedly
☐	☒	The statistical test(s) used AND whether they are one- or two-sided<br>*Only common tests should be described solely by name; describe more complex techniques in the Methods section.*
☒	☐	A description of all covariates tested
☐	☒	A description of any assumptions or corrections, such as tests of normality and adjustment for multiple comparisons
☐	☒	A full description of the statistical parameters including central tendency (e.g. means) or other basic estimates (e.g. regression coefficient) AND variation (e.g. standard deviation) or associated estimates of uncertainty (e.g. confidence intervals)
☐	☒	For null hypothesis testing, the test statistic (e.g. *F*, *t*, *r*) with confidence intervals, effect sizes, degrees of freedom and *P* value noted<br>*Give P values as exact values whenever suitable.*
☒	☐	For Bayesian analysis, information on the choice of priors and Markov chain Monte Carlo settings
☒	☐	For hierarchical and complex designs, identification of the appropriate level for tests and full reporting of outcomes
☒	☐	Estimates of effect sizes (e.g. Cohen's *d*, Pearson's *r*), indicating how they were calculated

Our web collection on statistics for biologists contains articles on many of the points above.

Software and code

Policy information about availability of computer code

Data collection	Histology: Hamamatsu NanoZoomer Digital Slide Scanners, Akoya Vectra Polaris Multispectral Imaging and Whole Slide Scanning<br>real-time PCR: StepOne software (v2.3)<br>Bioinformatics: For RNA sequencing of mouse cohort, FASTQ files quality was assessed using FastQC (v0.11.9), sequencing adapter content was trimmed using FastP (v0.20.0). The pre-processed reads were aligned to the mouse GRCm38/mm10 reference genome using STAR (v2.7.3a). Gene mapping and quantification was performed using TEtranscripts (v2.1.4). For microarray data of mouse cohort, Limma (v3.50.1) was used to read raw text files from single-channel Agilent RNA microarrays. For publicly available datasets, TCGA-COAD/READ count matrices were accessed using TCGAbiolinks (v2.24.3). No specialized software was used for other public datasets - see Method section of the manuscript for details.
Data analysis	We used GraphPad Prism (v9.4.0), QPath (v0.4.2), Phenochart (v1.0.12), Inform software, StepOne software (v2.3).<br>For bioinformatics: All software used in this study are published and cited in Method section of the manuscript. Here is the list of software used in this study for data processing and analysis: R (v3.6.1 & v4.0.5 & v4.1.0), FastQC (v0.11.9 & v0.11.5), FastP (v0.20.0), STAR (v2.7.3a & v2.7.9a) , TEtranscripts (v2.1.4), DESeq2 (v1.34.0 & v1.36.0), limma (v3.50.1), BioMart (v2.50.3), clusterProfiler (v4.4.2), enrich plot (v1.14.2), TCGAbiolinks (v2.24.3), GSEA software (v4.2.2), Python (v3.9.1), Anndata (v0.7.6), pandas (v1.3.1), Seurat (v3.2.3 & v4.1.1), UCell (v1.3.1), ggpubr (v0.4.0), pySCENIC (v0.11.2), GRNBoost2 (arboreto, v0.1.6), Scanpy (v1.9.1), iRegulon (v1.3), Cytoscape (v 3.9.1)

For manuscripts utilizing custom algorithms or software that are central to the research but not yet described in published literature, software must be made available to editors and reviewers. We strongly encourage code deposition in a community repository (e.g. GitHub). See the Nature Portfolio guidelines for submitting code & software for further information.

Data

Policy information about availability of data

All manuscripts must include a data availability statement. This statement should provide the following information, where applicable:

- Accession codes, unique identifiers, or web links for publicly available datasets
- A description of any restrictions on data availability
- For clinical datasets or third party data, please ensure that the statement adheres to our policy

Provide your data availability statement here.

Human research participants

Policy information about studies involving human research participants and Sex and Gender in Research.

Reporting on sex and gender	N/A
Population characteristics	N/A
Recruitment	N/A
Ethics oversight	N/A

Note that full information on the approval of the study protocol must also be provided in the manuscript.

Field-specific reporting

Please select the one below that is the best fit for your research. If you are not sure, read the appropriate sections before making your selection.

☒ Life sciences ☐ Behavioural & social sciences ☐ Ecological, evolutionary & environmental sciences

For a reference copy of the document with all sections, see nature.com/documents/nr-reporting-summary-flat.pdf

Life sciences study design

All studies must disclose on these points even when the disclosure is negative.

Sample size	Sample size was not calculated for these experiments
Data exclusions	No data were excluded from analysis
Replication	All experiments included biological replicates as indicated in the methods or figure legends. Several findings were also confirmed using orthogonal experimental approaches.
Randomization	Mice were randomly allocated to treatment or vehicle control following genotyping results. however, allocation was such that an equal number of male and females was allocated in each group with transcriptomic data. For statin treatment experiment, complete randomization was employed as only females animals were used.
Blinding	Data scoring was performed by blinding the scorer to the identity of the samples or the identity of different groups of animals. Data were unblinded and analysed after scoring. In some experiments (e.g. RNAseq, western blotting and real-time PCR) blinding was not possible or not practical. However, those methodologies are objective and blinding is not necessary.

Reporting for specific materials, systems and methods

We require information from authors about some types of materials, experimental systems and methods used in many studies. Here, indicate whether each material, system or method listed is relevant to your study. If you are not sure if a list item applies to your research, read the appropriate section before selecting a response.

Materials & experimental systems

n/a	Involved in the study
☐	☒ Antibodies
☒	☐ Eukaryotic cell lines
☒	☐ Palaeontology and archaeology
☐	☒ Animals and other organisms
☒	☐ Clinical data
☒	☐ Dual use research of concern

Methods

n/a	Involved in the study
☒	☐ ChIP-seq
☒	☐ Flow cytometry
☒	☐ MRI-based neuroimaging

Antibodies

Antibodies used

Lysozyme, DAKO, A0099
OLFM4, Cell Signalling, 39141
Chromogranin A, Immunostar, 20085
BrdU, Cell Signalling, 5292
Cleaved-PARP, Cell Signalling, 94885
p-ERK1/2, Cell Signaling, 9101S
ERK2, Santa Cruz, sc-1647

Validation

Antibody validation was from the respective vendors:
https://www.agilent.com/store/productDetail.jsp?catalogId=A009902-2
https://www.cellsignal.com/products/primary-antibodies/olfm4-d6y5a-xp-rabbit-mab/39141
https://www.immunostar.com/product/sp-1-chromogranin-a-bovine-antibody/
https://www.cellsignal.com/products/primary-antibodies/brdu-bu20a-mouse-mab/5292
https://www.cellsignal.com/products/primary-antibodies/cleaved-parp-asp214-d6x6x-rabbit-mab/94885
https://www.cellsignal.com/products/primary-antibodies/phospho-p44-42-mapk-erk1-2-thr202-tyr204-antibody/9101
https://www.scbt.com/p/erk-2-antibody-d-2

Animals and other research organisms

Policy information about studies involving animals; ARRIVE guidelines recommended for reporting animal research, and Sex and Gender in Research

Laboratory animals

Mus Musculus, C57BL6, up to 6 months of age

Wild animals

Provide details on animals observed in or captured in the field; report species and age where possible. Describe how animals were caught and transported and what happened to captive animals after the study (if killed, explain why and describe method; if released, say where and when) OR state that the study did not involve wild animals.

Reporting on sex

All findings were obtained on both sexes, with the exception of the stating treatment experiment in vivo, which was obtained using female animals.

Field-collected samples

For laboratory work with field-collected samples, describe all relevant parameters such as housing, maintenance, temperature, photoperiod and end-of-experiment protocol OR state that the study did not involve samples collected from the field.

Ethics oversight

All animal experiments were performed according to Home Office guidelines under project licenses (PPL) PC4E1710A and P7B8067BB. Animal experiments were approved by the local ethics committee at the University of Leicester..

Note that full information on the approval of the study protocol must also be provided in the manuscript.
